# Organochlorinated pesticides expedite the enzymatic degradation of DNA

**DOI:** 10.1038/s42003-019-0326-5

**Published:** 2019-02-26

**Authors:** Chao Qin, Bing Yang, Wei Zhang, Wanting Ling, Cun Liu, Juan Liu, Xu Li, Yanzheng Gao

**Affiliations:** 1 Institute of Organic Contaminant Control and Soil Remediation, College of Resources and Environmental Sciences, Nanjing Agricultural University, 210095 Nanjing, China; 2 Department of Plant, Soil and Microbial Sciences, and Environmental Science and Policy Program, Michigan State University, East Lansing, MI 48824 USA; 3 Key Laboratory of Soil Environment and Pollution Remediation, Institute of Soil Science, Chinese Academy of Sciences, 210023 Nanjing, China; 4 Department of Civil Engineering, University of Nebraska–Lincoln, Lincoln, NE 68588 USA

## Abstract

Extracellular DNA in the environment may play important roles in genetic diversity and biological evolution. However, the influence of environmental persistent organic contaminants such as organochlorinated pesticides (e.g., hexachlorocyclohexanes [HCHs]) on the enzymatic degradation of extracellular DNA has not been elucidated. In this study, we observed expedited enzymatic degradation of extracellular DNA in the presence of α-HCH, β-HCH and γ-HCH. The HCH-expedited DNA degradation was not due to increased deoxyribonuclease I (DNase I) activity. Our spectroscopic and computational results indicate that HCHs bound to DNA bases (most likely guanine) via Van der Waals forces and halogen bonds. This binding increased the helicity and accumulation of DNA base pairs, leading to a more compact DNA structure that exposed more sites susceptible to DNase I and thus expedited DNA degradation. This study provided insight into the genotoxicity and ecotoxicity of pesticides and improved our understanding of DNA persistence in contaminated environments.

## Abstract (web summary)

Chao Qin, Bing Yang et al. show that organochlorinated pesticides as environmentally persistent organic contaminants enhance the degradation of extracellular DNA by making it more accessible to DNase I. This study provides insight into the persistence of DNA in contaminated environments.

## Introduction

Genetic diversity is the basis for evolution and the differentiation of species on Earth. Genetic mutation, recombination, and transformation are the driving factors in genetic diversity. Previous studies have primarily focused on in vivo DNA due to its recognized importance in biological evolution, diversity, and toxicity, whereas studies on environmental and biological behaviors of extracellular DNA have been scarce. Extracellular DNA released from prokaryotic and eukaryotic cells is the largest fraction of total environmental DNA^1^ and has been detected in various environmental compartments such as marine water (568–3163 ng mL^−1^)^2^ and freshwater (9–11 ng mL^−1^)^3,4^. Extracellular DNA can be excreted, degraded, or taken up as a nutrient source by microorganisms. It can also be sorbed onto minerals, thus promoting its environmental persistence^5^ and potentially preserving genetic information from the past. DNA residues in the environment can interact with other contaminants, and thus change environmental behaviors of both DNA and contaminants. The binding of plasmid DNA to polycyclic aromatic hydrocarbons decreases its transformation efficacy^6^. Thus, it is important to study the interactions of DNA with other contaminants and associated effects of these interactions on the fate of extracellular DNA in the environment.

The environmental abundance and biological significance of extracellular DNA are primarily controlled by its degradation. DNA can be degraded via hydrolysis, oxidation, and enzymatic reaction^7–9^, and the degradation products (nucleotides and nucleosides) can be re-assimilated by microorganisms. For example, Fe(II)∙bleomycin can cause the O_2_-dependent DNA hydrolysis, starting with the fracture of the deoxyribose 3’-4’-carbon bond, and finally the breakage of DNA into oligonucleotides, bases, and compounds resembling malondialdehyde^10^. DNA can also be oxidized by oxidants such as reactive oxygen species. Ozone can damage DNA directly and indirectly by degrading base and sugar moiety with hydroxyl radicals^11^. Nonetheless, enzymatic reaction is considered as the main degradation pathway of DNA in the environment^12,13^. In fact, the DNA degradation is largely controlled by the species, activities, and reaction modes of DNA-degrading enzymes^14^. Among the DNA-degrading enzymes, homing endonucleases are double-stranded DNase that attack large recognition sites (12–40 bp) of DNA by making a site-specific double-strand breakage at a target site in an allele free of the corresponding mobile intron^15^. Microbial restriction endonuclease I can cleave DNA into smaller duplex DNA fragments of about 400-bp oligonucleotides^16^. DNase I can cleave the phosphodiester backbone of the DNA double helix in the presence of divalent cations (e.g., Mg^2+^ and Ca^2+^), and introduce single-stranded nicks through hydrolysis of the P-O_3_’-bond, resulting in 5’-phosphorylated fragments^17^.

It is well known that the enzymatic degradation of DNA is dependent on environmental factors such as solution pH, and the type and concentration of cations.^18^ The activity of DNase I is the highest at approximately pH 7.0 in the presence of Mg^2+^ and Ca^2+^.^19,20^ The proton acceptor–donor chain E-H-water of DNase I is essential to the DNA degradation.^21^ Briefly, the carboxylate anion of E 75 can accept a proton from H 131, which in turn receives a proton donated by a water molecule. The resultant reactive water hydroxyl can then initiate the nucleophilic attack of the phosphorus atom, and thus cleave the P-O-3’ bond. During this reaction, the Ca^2+^ ion can facilitate the nucleophilic attack by properly aligning the phosphodiester bond to DNase I. Moreover, at acidic solution pH, the H 131 can be protonated and is then unable to accept a proton from a water molecule, leading to the inactivation of DNase I. Some organic molecules can also affect DNA degradation^22,23^. For example, due to an unknown mechanism, DNA bound to humic acid was less susceptible to DNase I degradation than free DNA^24^. Neomycin B (an aminoglycoside antibiotic) completely inhibits DNA degradation by DNase I in vitro at a concentration of 2 mmol L^−1^ ^25^, due to the conformational transition from B-DNA to A-DNA induced by the binding of neomycin to DNA^26^. Considering that a myriad of synthetic organic compounds has been released into the environment by human activities, it is of great interest to study the effect of some representative compounds on the enzymatic degradation of DNA. Pesticides deserve a particular attention because of their extensive use, their environmental persistence, and bioaccumulation. This study focused on hexachlorocyclohexanes (HCHs) that are broad-spectrum insecticides. This group of pesticides was widely produced and used from the early 1950s to the late 1980s, resulting in their ubiquitousness in the environment and their bioaccumulation through the food chain. China and India, the two primary users of HCH, stopped its agricultural use in 1983 and 1990, respectively. However, HCH was still used in India until 1995 with a peak use of 25,000 tons during the 1990s^27^. Although HCH has been banned, the legacy HCH present in the environment from the previous uses are very persistent and can still have adverse effects on human and ecosystem health^28^. Besides, in terms of structure, HCHs have no other elements and functional groups except C and H elements, which highlight the role of chlorine. Three HCH isomers (α-HCH, β-HCH, and γ-HCH) with varying physicochemical properties and biological activities were studied, likely due to the changing chiral arrangement of chlorine atoms on the cyclohexane ring^29^. Nonetheless, the influence of HCHs on DNA environmental behavior has been rarely studied.^30^ Thus, HCHs are ideal candidates for examining the effect of organochlorinated contaminants on the enzymatic degradation of DNA.

Therefore, we aimed to elucidate the effects, and underlying mechanism, of three HCH isomers on the enzymatic degradation of DNA. DNA degradation by DNase I in the presence of HCH was examined by gel electrophoresis, followed by a series of spectroscopic and computational investigations to pinpoint the underlying mechanisms. The change of DNase I activity upon HCH exposure was examined spectroscopically, whereas fluorescence quenching titration experiments were conducted to determine the binding of HCH with DNA. The binding mechanism and resultant conformational change of DNA structure were probed by molecular computation, Fourier transformed infrared spectroscopy (FTIR), ultraviolet (UV)-Vis spectroscopy, and circular dichroism (CD). Our results provided insight into the enzymatic degradation of DNA as influenced by organochlorinated contaminants, thus improving understanding of the behaviors of DNA in contaminated environments.

## Results

### HCHs expedited enzymatic degradation of DNA

**Fig. 1:**
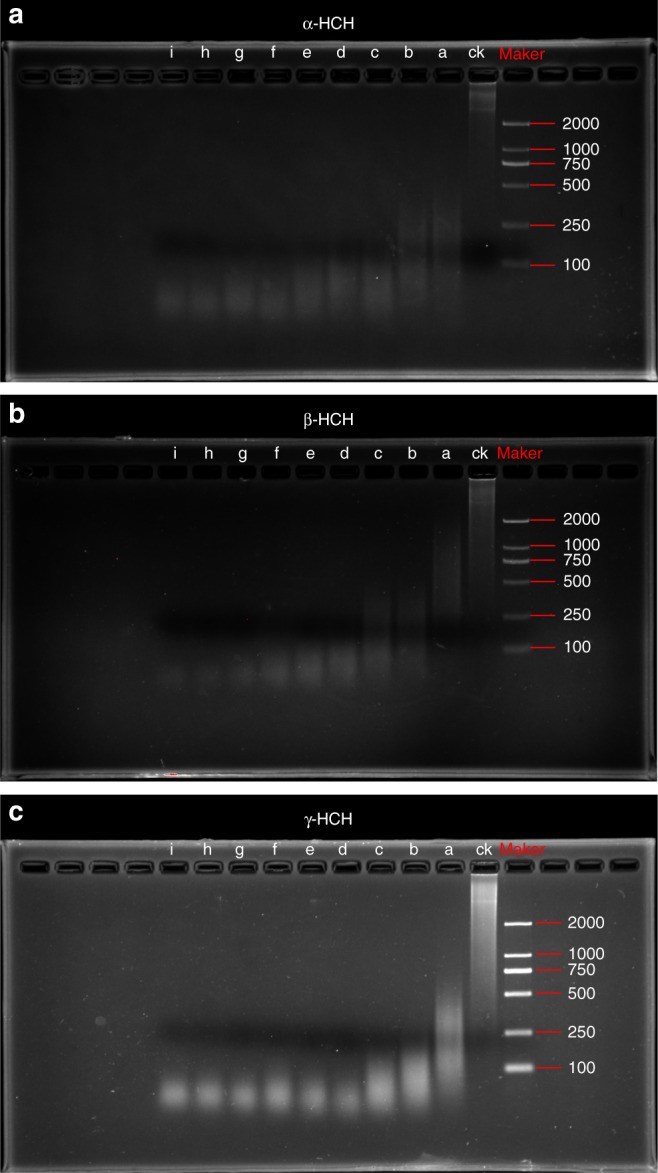
Gel electrophoresis of DNA fragments. **a** α-HCH, **b** β-HCH, and **c** γ-HCH (0–4.0 mg L^−1^). a, b, c, d, e, f, g, h, and i represent hexachlorocyclohexane (HCH) concentrations of 0, 0.5, 1.0, 1.5, 2.0, 2.5, 3.0, 3.5, and 4.0 mg L^−1^, respectively. Ck, control treatment without DNase I

**Fig. 2:**
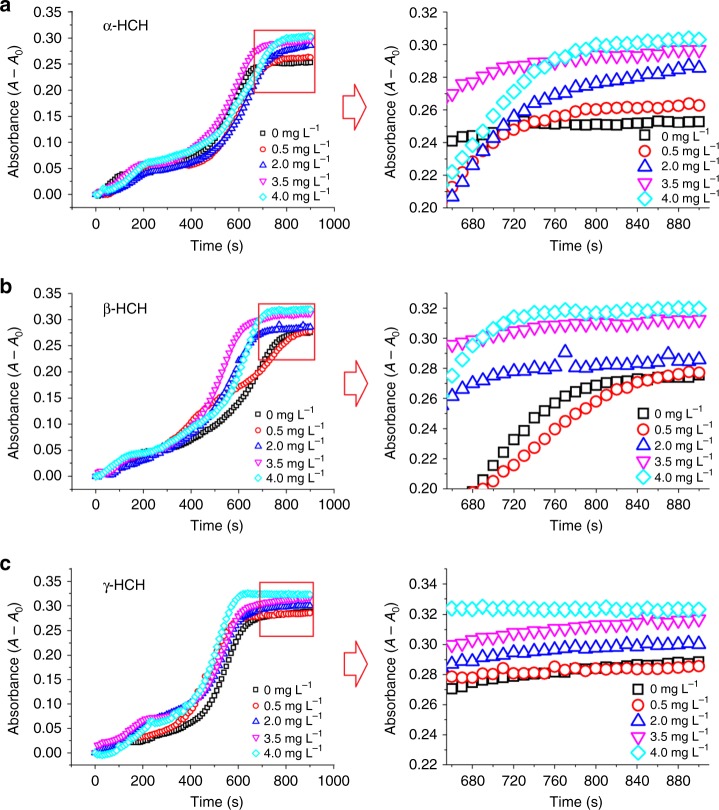
Increase of DNA absorbance caused by DNase I in the presence of hexachlorocyclohexanes (HCHs). **a** The DNA absorbance changes influenced by α-HCH. **b** The DNA absorbance changes influenced by β-HCH. **c** The DNA absorbance changes influenced by γ-HCH. Each absorbance data point was the average of 10 measurements

In the absence of DNase I, most DNA fragments were larger (~2000 bp), and there were no fragments smaller than 100 bp (Fig. 1). However, in the presence of DNase I, DNA was degraded into smaller fragments, the size of which decreased from 2000 bp to <100 bp with increasing HCH concentrations (Figs. 1a–c). The HCH-mediated increase in DNA degradation was corroborated by the absorbance values (Fig. 2). The final absorbance of the DNA solutions generally increased with the HCH concentration (Fig. 2). The increase in the absorbance of the DNA solution was due to greater exposure of nitrogenous nucleobases to light incidence, most likely resulted from the disassembly of DNA structure. To our knowledge, this is the first report that a pesticide promotes enzymatic degradation of DNA. As discussed previously, DNase I cleaves the phosphodiester bond using the reactive water hydroxyl formed by the proton acceptor–donor E-H-water chain, and thus better alignment of the phosphodiester bond to DNase I could enhance DNA degradation.^21^ The increased DNase I-catalyzed degradation of DNA by HCHs was likely because the binding of HCHs to DNA improves the alignment of the phosphodiester bond to the nucleophiles (water hydroxyls), enhancing nucleophilic attack of the phosphorus atom, or because HCHs bound to DNase, I increased its activity.

### Effects of HCH on DNase I activity

**Fig. 3:**
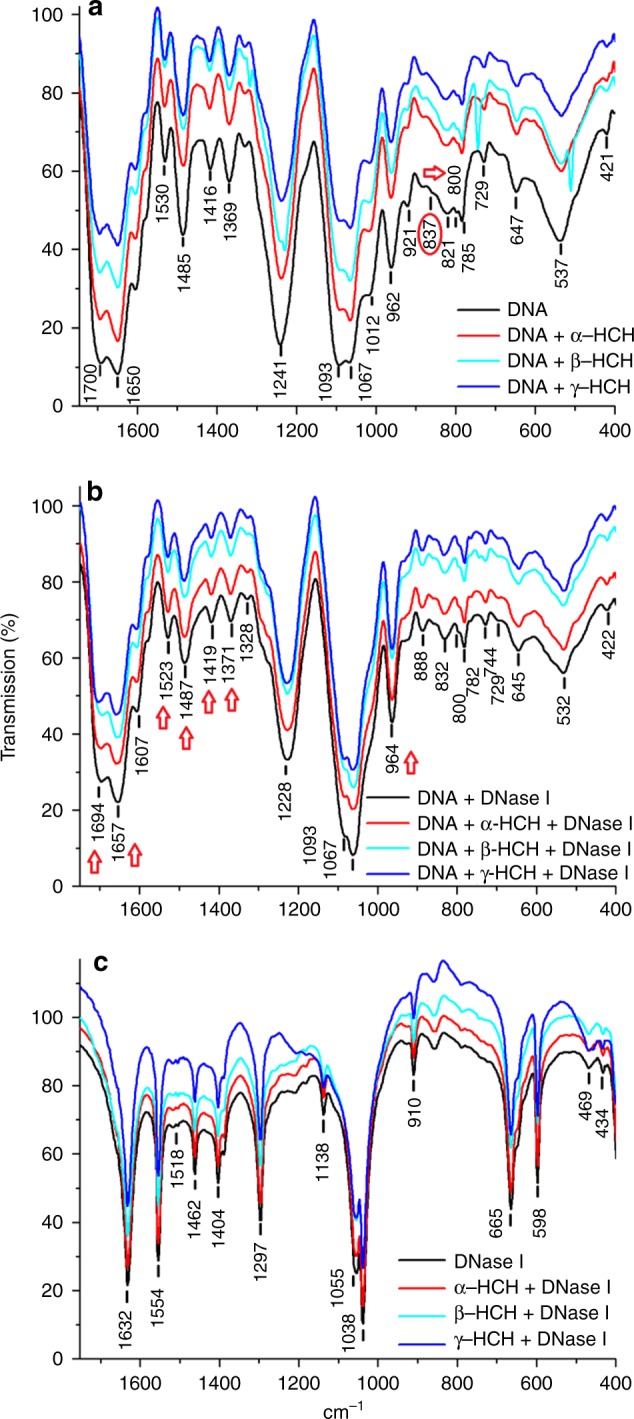
FTIR spectra of DNA, DNA–hexachlorocyclohexane (HCH), DNA–HCH–DNase I, and HCH–DNase I. FTIR spectra of **a** DNA in the absence or presence of HCHs (4.0 mg L^−1^), **b** DNA mixed with HCH and DNase I, and **c** DNase I mixed with HCHs. FTIR Fourier transformed infrared spectroscopy

To clarify the mechanism of HCH-expedited enzymatic degradation of DNA, we first examined the influence of three HCHs on the DNase I activity by the spectrophotometric experiments. The activity of DNase I decreased from 1.43 to 0.80 U as the α-HCH concentration increased from 0 to 2.5 mg L^−1^ and subsequently increased to 1.49 U (Supplementary Figure 1). β-HCH or γ-HCH did not affect the enzymatic activity of DNase I. Higher Log *K*_ow_ of α-HCH (3.8) probably perturb the activities of DNase I than β-HCH (3.78) and γ-HCH (3.72)^29^, which means more insoluble α-HCH is easier to bind to DNase I or DNA, thereby affecting the activity of the DNase I. Thus, the three HCH isomers did not enhance the activity of DNase I. Additionally, as revealed by the FTIR spectra (Fig. 3), the structure and functional groups of DNase I did not change in the presence of HCHs, again indicating no change in the enzymatic activity.^31^ No shifts were observed in the bands near 1632 cm^−1^ for amide I (C (N) = O), the bands near 1554 cm^−1^ and 1462 cm^−1^ for amide II (C-N + N-H), and the bands near 1404 cm^−1^ and 1297 cm^−1^ for amide III (N-H bending and C-N stretching vibrations) in the presence compared with the absence of HCHs (Fig. 3c). Therefore, the increased DNA degradation could not be attributed to expedited DNase I activity. Interestingly, *N*-bromosuccinimide reportedly inhibits the activity of DNase II^32^. However, few other studies have addressed the effect of exogenous organic pollutants on DNase I activity. To our knowledge, this is the first study on the influence of an organic contaminant on DNase I activity.

### Binding of HCHs to DNA

**Fig. 4:**
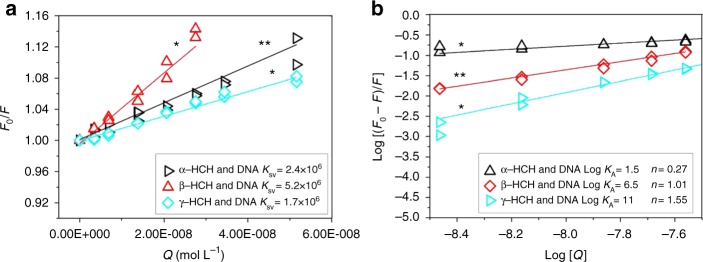
Binding of hexachlorocyclohexanes (HCHs) with ethidium-bromide-labeled DNA probed by the fluorescence quenching. **a** Stern–Volmer plot. **b** Plot of Log [(*F*_0_ − *F*)/*F*] vs Log [*Q*]. DNA = 10 mg L^−1^, HCH = 0–15 μg L^−1^, ethidium-bromide = 2 mg L^−1^, and pH = 7.0. **p < 0.01, *p < 0.05

DNA is a vital biomacromolecule that can bind to other molecules or ions^33^. The binding can often change the structures and functions of DNA. It is likely that the binding of HCHs to DNA may be responsible for expedited DNA degradation. The binding of HCHs to DNA was first confirmed by fluorescence quenching of ethidium-bromide-labeled DNA (Fig. 4). The quenching constant (*K*_SV_) calculated from the Stern–Volmer plot (Fig. 4a) is commonly used to quantify the quenching effect. The average *K*_SV_ values for α-HCH, β-HCH, and γ-HCH were 2.4 × 10^6^, 5.2 × 10^6^, and 1.7 × 10^6^ L mol^−1^, respectively. The quenching constant for the γ-HCH is smaller than others and this result is consistent with the calculated binding energy (Supplementary Table 1). The bimolecular quenching rate constant (*K*_q_) indicates the stability of binding between DNA and HCHs. The *K*_q_ values for α-HCH, β-HCH, and γ-HCH were estimated to be (0.2–6.0) × 10^14^, (0.4–13.0) × 10^14^, and (0.1–4.3) × 10^13^ L mol^−1^ s^−1^, respectively. Furthermore, a linear relationship between log [(*F*_0_* − F*)*/F*] and log [*Q*] (*R*^2 ^> 0.99), suggesting a static quenching process (Fig. 4b). The binding constant (*K*_A_) indicates the binding strength, and the number of binding sites (*n*) suggests a dose–ratio relationship of the binding. The *K*_A_ values were 1.5, 6.5, and 11.0 L mol^−1^, and the *n* values were 0.27, 1.01, and 1.55 for α-HCH, β-HCH, and γ-HCH, respectively. Thus, the three HCH isomers can bind to DNA.

Moreover, HCH binding to DNA changed the DNA structure, because the absorption bands of the DNA–HCH complexes near 1700, 1650, 1530, 1485, 1416, 1369, and 962 cm^−1^ were weaker than those of DNA (Fig. 3a). The weakened absorption bands were assigned to guanine carbonyl vibration, C=C or C=N in base stretch, an imidazole ring, DNA structural vibration, and guanine, respectively (Supplementary Table 2). A red shift occurred on the FTIR bands at 1487, 1419, 1371, and 964 cm^−1^, representing DNA structural and backbone vibrations, guanine, and asymmetric PO_2_^−^ (Figs. 3a, b). Additionally, the thymine band at 1650 cm^−1^ and the amide I of DNase I at 1647 cm^−1^ may overlap at specific enzyme concentrations. However, the presence of the strong positive feature at 1657 cm^−1^ (Fig. 3b) cannot be attributed to the overlap of absorption bands of thymine or protein amide I. It is more likely due to a stretching change in C=C or C=N in the DNA bases. Therefore, the FTIR analyses indicated that the DNA bases were the most probable sites for binding of HCHs.

**Fig. 5:**
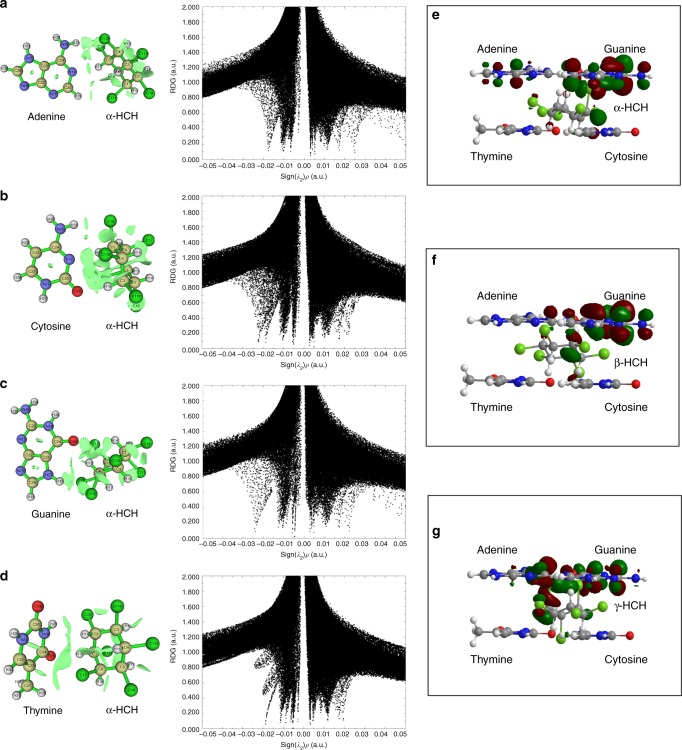
Computational results of hexachlorocyclohexane (HCH) binding to bases. Representative image of the gradient isosurface (left), the corresponding plots of reduced density gradient versus the sign of the second Hessian eigenvalues (right) of **a** adenine–α-HCH, **b** cytosine–α-HCH, **c** guanine–α-HCH, **d** thymine–α-HCH, together with the molecular orbitals of **e** DNA bases–α-HCH, **f** DNA bases–β-HCH, and **g** DNA bases–γ-HCH. The surfaces are colored on a blue–green–red scale according to the sign(*λ*_2_)*ρ* values (range −0.05 to 0.05 a.u.). Green areas between molecules indicate a weak Van der Waals force. Large brown and olive spheres represent the positive and negative phases, respectively, of the electronic wave function

Many organic chemicals such as phenothiazinium dyes and polycyclic aromatic hydrocarbons can be inserted into double-stranded DNA^9,34^. Actually, we recently reported that a π–π isosurface between the DNA bases and polycyclic aromatic hydrocarbons may be responsible for their binding^9^. However, the π–π interactions cannot explain the binding of HCHs to DNA, due to the lack of conjugated groups in the HCH structure. To explore other weak interaction forces that potentially mediate the binding of HCHs to DNA, the intermolecular forces between DNA bases and HCHs were simulated. The electrostatic potentials of the four DNA bases (adenine, thymine, guanine, and cytosine) and HCH isomers are shown in Supporting Information (Supplementary Figure 2). During the reduced density gradient analysis, the isosurface color based on the values of sign(*λ*_2_)*ρ* (Supplementary Figure 3) was used to explain the multiple weak attractions between the DNA bases and HCHs. In the blue region (*ρ* > 0, and *λ*_2_ < 0), the smaller value of *ρ* means the stronger attraction. In this region, the most attractive force is hydrogen bonding or halogen bonding. The green region (*ρ* ≈ 0, and *λ*_2_ ≈ 0) is very small, suggesting a weak interaction consistent with the Van der Waals force. Since the electron density of this region is very small, the sign of *λ*_2_ becomes more unstable and can be negative. In the red region (*ρ* > 0, and *λ*_2_ > 0), the greater value of *ρ* means the stronger mutual exclusion is corresponding to the strong steric effect region (also called the nonbonded overlap) at the benzene rings. The bright green isosurface between molecules indicates weak Van der Waals force between the DNA base and α-HCH (Figs. 5a–d). The low-gradient spikes at –0.005 to –0.015 a.u. show the Van der Waals interaction patterns between the DNA bases and α-HCH. There was also a greater negative value at –0.02 a.u. (equivalent to the strength of H-bonds^35^), representing strong halogen bonding of α-HCH to the DNA bases. Similar results were obtained for the binding of DNA bases to β-HCH (Supplementary Figure 4) and γ-HCH (Supplementary Figure 5). These data confirmed insertion of HCHs into the DNA double strands and their binding to the DNA bases via Van der Waals forces and halogen bonds. The halogen bonding of HCH to the DNA bases indicates that other organochlorinated compounds may share a similar interaction with DNA and thus also enhance its enzymatic degradation. The highest occupied molecular orbital and lowest unoccupied molecular orbital between DNA bases and HCHs analyzed by ChemBioOffice2010 (Figs. 5e–g). The positive and negative phases of the electronic wave function are represented by larger brown and green spheres, respectively. Evidently, guanine has a higher electron cloud density than those of the other DNA bases and is, therefore, more likely to bind to HCHs.

### DNA deformation

**Fig. 6:**
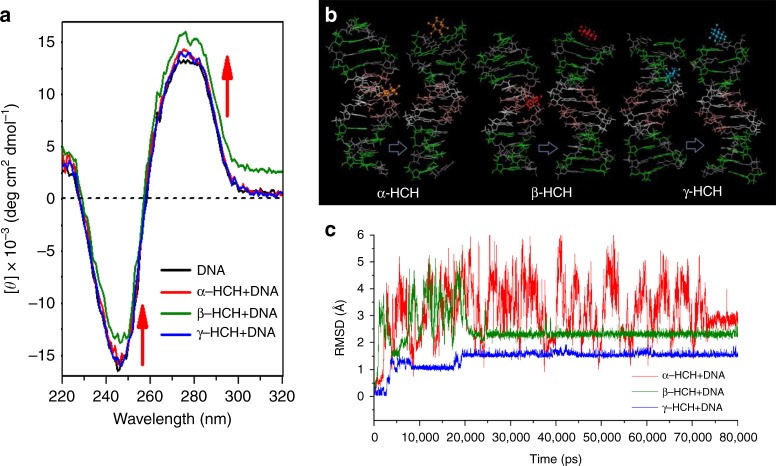
Structural changes in DNA and stability of DNA–hexachlorocyclohexane (HCH) complexes. **a** Circular dichroism spectra of DNA (black line), α-HCH–DNA (red line), β-HCH–DNA (olive line), and γ-HCH–DNA (blue line). Red arrow indicates a shift in molar ellipticity. **b** Optimized DNA–HCH structure. **c** RMSD over 80,000 ps α-HCH–DNA (red line), β-HCH–DNA (olive line), and γ-HCH–DNA (blue line)

Next, we analyzed whether the insertion of HCH into the DNA double strands could change the DNA conformation. It is hypothesized that the change of DNA conformation in the HCH presence may enhance the DNA degradation by DNase I. Based on the UV-Vis spectra, the changes of the characteristic absorption band of DNA at 260 nm^36^ were examined in the presence of HCHs at various concentrations (Supplementary Figure 6). It was reported that with increasing concentrations of metal cations the increase of absorbance at 260 nm (i.e., hyperchromism) suggests the damage or distortion of the DNA double helix, whereas the decrease of absorbance (i.e., hypochromism) likely resulted from the DNA contraction along the helix axis and the conformational change of DNA.^36^ The decreased absorbance at 260 nm with increasing HCH concentration suggests that the binding of HCHs with DNA resulted in the changes in the DNA conformation. These conformational changes of DNA were further supported by the CD spectra analyses (Fig. 6a). The CD spectrum of DNA consists of a negative Cotton effect at 248 nm and a positive Cotton effect at 276 nm. As previously reported^37^, the negative spectrum corresponds to the helical structure of DNA, and the positive spectrum represents the accumulation of base pairs that is characteristic of DNA in the right-handed B-form. HCH binding caused an increase in the intensity of both positive and negative bands without a shift in the peak positions (Fig. 6a), indicating increases in both helicity and base pair accumulation. More importantly, these changes may result in a more compact DNA structure and exposure of more DNA sites susceptible to DNase I, thus promoting DNA degradation. In contrast, the binding of neomycin or polycyclic aromatic hydrocarbons to DNA caused a transition from B-DNA to A-DNA, leading to inaccessibility of the minor groove (a smaller binding area for DNase I) and consequently inhibition of DNA degradation^25^. Additionally, comparing with α-HCH–DNA and γ-HCH–DNA, the CD spectra change of β-HCH–DNA is larger. It is probably because all chlorine atoms in the β-HCH molecule are in axial and equatorial positions in each HCH molecule, giving this isomer larger chemical stability. Molecular dynamics simulation from AMBER17 was performed for the best poses selected from the docking studies (Fig. 6b). After the initial equilibration, molecular dynamics production run was carried out for 80 ns. The root-mean-square deviation (RMSD) as a function of time for all the complexes was plotted (Fig. 6c) to assess their systematic deviation. The range of RMSDs (Fig. 6c) is 0.5 Å–6.0 Å. Considering subtle changes in the helicoidal structure, the RMSDs indicate that the structure is considerably converged. Convergence of RMSD values is indicative of the stability of a complex^38^. The α-HCH–DNA complexes were more active than β-HCH–DNA and γ-HCH–DNA during the simulation. This result indicates that β-HCH and γ-HCH binding with DNA led to a more stable converging than α-HCH.

### Conclusions

In this study, we attempted to identify mechanisms responsible for the expedited DNA degradation by DNase I in the presence of HCH. The increased enzymatic activity of DNase I, in the presence of HCHs, was excluded as a possible cause of the expedited DNA degradation. HCHs first binds to the bases (most likely guanine) of DNA via Van der Waals forces and halogen bonds, which caused the increases in helicity and base pair accumulation, as well as a more compacted structure of DNA exposing more sites susceptible to DNase I. This investigation provided insights into the enzymatic degradation of DNA in the environment, as influenced by HCHs. It also presents a new view on the genotoxicity and ecotoxicity of pesticides in the environment. Our results suggest that the legacy pesticides such as organochlorinated pesticides may decrease the amount of residual DNA in our environment by enhancing the DNA degradation. This study showed that combining experimental observations with molecular computation on interactions of DNA and contaminants could be a useful approach for understanding the environmental behaviors and risks of both DNA and contaminants. Future studies could be directed to other kinds of DNA causing major environmental concerns (e.g., antibiotic resistance genes), and other types of persistent organic pollutants (e.g., polycyclic aromatic hydrocarbons and polybrominated biphenyls) to improve our understanding on environmental behaviors of DNA in a contaminated environment.

## Methods

### Chemicals

Salmon sperm DNA with an average molar mass of 1.3 × 10^6^ Da and %G-C content of 41.2%, α-HCH, β-HCH, and γ-HCH were purchased from Sigma Chemical Co. (St. Louis, MO, USA). DNase I and DNase I buffer (10 × ) were purchased from Takara Bio Company (Dalian, Liaoning, China). The physicochemical properties of α-HCH, β-HCH, and γ-HCH are given in Supplementary Table 4. All chemical reagents were of analytical grade and used without further purification. DNA stock solution of 1 g L^−1^ was prepared by dissolving 0.1 g DNA in 100 mL Tris-HCl buffer (10 mmol L^−1^, pH 7.0). Stock solutions of α-HCH, β-HCH, or γ-HCH (1 g L^−1^) were prepared using methanol as solvent. HCH working solutions were prepared by further diluting their stock solution by methanol to 10 mg L^−1^. All solutions were stored at 4 °C in a refrigerator before use.

### DNA degradation by DNase I

We performed gel electrophoresis to evaluate DNA degradation. First, 10 μL of the DNase I buffer (10 × ) was pipetted into a series of PCR tubes. Next, a predetermined volume of the working solution of α-HCH, β-HCH, or γ-HCH (10 mg L^−1^) was added to each tube to obtain a series of HCH concentrations (0, 0.5, 1.0, 1.5, 2.0, 2.5, 3.0, 3.5, or 4.0 mg L^−1^), respectively, followed by adding 10 μL of the 1 g L^−1^ DNA stock solution. The tubes were filled with the Tris-HCl buffer to a volume of 99 μL and then incubated on ice for 120 min. Then, the tubes were transferred to room temperature, and 1 μL of the DNase I working solution was added to the tubes. The mixture in the tubes were homogenized and then incubated for 20 min at 37 °C. Afterwards, 10 μL aliquots were taken from each tube, mixed with 10 μL of the loading buffer containing 30 mM EDTA, 50% (v/v) glycerol, 0.25% (w/v) Xylene Cyanol FF, and 0.25% (w/v) Bromophenol Blue (QsingKe Biological Technology, Nanjing, China), and then loaded onto the agarose gel (3% w/v). The gel electrophoresis was run at 6 V cm^−1^ for 1 h. The DL2000 DNA ladder (80 ng μL^−1^) (Takara Bio Company, Dalian, China) was used as a marker in the gel electrophoresis. A Bio-Rad Molecular Imager FX (Hercules, Canada) equipped with the appropriate excitation and emission standard filters were used to image the labeled DNA bands. The gel was then stained with ethidium-bromide (final concentration, 0.5 μg mL^−1^) for 30 min and visualized by the Bio-Rad Quantity One software (Hercules, Canada). The experiment results were repeated three times.

### Enzymatic degradation kinetics and DNase I activity

The enzyme activity of DNase I was examined by spectrophotometric experiments. First, in each PCR tube, 10 μL of the DNase buffer (10 × ) was mixed with 10 μL of the 1 g L^−1^ DNA stock solution. Then 0, 5, 10, 15, 20, 25, 30, 35, or 40 μL of α-HCH, β-HCH, or γ-HCH (10 mg L^−1^) were added to each tube to obtain the HCH concentration of 0, 0.5, 1.0, 1.5, 2.0, 2.5, 3.0, 3.5, or 4.0 mg L^−1^, respectively. These tubes were filled with the Tris-HCl buffer to a volume of 99 μL and then incubated on ice for 120 min. Next, the 100 μL mixture in the tubes were transferred to 96-well enzyme-linked immunosorbent assay (ELISA) plate and incubated for 20 min at 37 °C. Then 1 μL of the DNase I working solution was added to the ELISA plate. The absorbance of DNA at 260 nm was recorded at 37 °C by an enzyme-labeled meter (SP-Max 2300A, Shanghai, China) over 15 min. Each absorbance data point was the average of 10 measurements. The DNase activity was calculated by the change of absorbance within the first minute via ${U = }\frac{{\Delta {A}}}{{V{\mathrm{ \times 0}}{\mathrm{.001}}}}$, where *U* is the enzymatic activity, $\Delta {A}$ is the absorbable change of DNA in the first minute, and *V* is the sample volume. The HCH solutions with the same concentration free of DNA and DNase I were also used as background solution to avoid the interference from each added HCH.

### Fluorescence quenching titration

Fluorescence quenching titration was performed to assess the binding of HCHs to DNA (Supplementary Methods).

### DNA conformation

The UV-visible spectra of the HCH and DNA mixtures were investigated using a Varian Cary 5000 Spectrophotometer (Cary 5000, Varian, Palo Alto, CA, USA). Briefly, 2 mL of a 100 mg L^−1^ DNA solution were added to a cuvette, and the UV spectrum from 245 to 275 nm was obtained at 25 ± 0.1 °C. Next, predetermined volumes of 1 g L^−1^ α-HCH, β-HCH, or γ-HCH stock solution were sequentially added to the cuvette at concentrations of 0–2400 μg L^−1^, 0–1000 μg L^−1^, and 0–10000 μg L^−1^, respectively. The solutions were stirred for 10 s before each HCH addition, followed by the acquisition of UV spectra. The pH value of the samples was 7.0 ± 0.2.

The CD spectrum was also acquired to examine DNA conformations. To prepare the DNA and HCH mixture at the most optimal DNA detection concentration of 60 mg L^−1^, 0.3 mL of 1 g L^−1^ DNA was mixed with 0.5 mL of 10 mg L^−1^ in each HCH, followed by the addition of 4.2 mL Tris-HCl buffer. Then, 2 mL aliquot was taken from each sample, placed into a rectangular quartz cuvette of 1 cm path length, and detected by a PC-driven JASCO J815 spectropolarimeter (Jasco International Co., Japan) with a temperature controller and a thermal programmer model PFD-425L/15. The CD spectra were recorded from 220 to 320 nm with a scan speed of 100 nm min^−1^ at 20 °C.

### Fourier transform infrared spectroscopy

Samples for FTIR analysis were prepared as described in Supplementary Methods. The IR absorption bands for the corresponding functional groups in DNA and DNase I are listed in Supplementary Table 2 and Supplementary Table 3, respectively.

### Molecular computation

Computational chemistry based on density functional theory was used to predict possible binding mechanism between HCH and DNA. Based on the FTIR analysis, we assumed that the most probable binding sites are the nitrogenous bases of DNA, as also suggested by our earlier work^9^. To decrease the computational load, we only constructed four nitrogenous bases (adenine, thymine, guanine, and cytosine) and α-HCH, β-HCH, and γ-HCH using GaussView 5.0^39^. The interactions of modeled bases with each HCH isomer was first optimized using the Gaussian 16 software at ωB97XD/6-311G** level. The structure and frequency were further analyzed using GAUSS at ωB97XD/6-311+G**. The Multiwfn program^40^ was used to analyze the results of gradient isosurface and related plots of reduced density gradient versus the electron density multiplied by the sign of the second Hessian eigenvalues. In fact, there are many methods for analyzing weak interactions, e.g., atoms in molecules topology analysis^41^, electrostatic potential analysis, and atomic charge analysis. Recently, Johnson et al. proposed that the reduced density gradient analysis is a beneficial method for analyzing weak interactions, which has gain popularity since then^35^. The reduced density gradient analysis can be considered the expansion of the atoms in molecules theory, and which can be applied to many cases when the original atoms in molecules theory is not applicable, e.g., π–π accumulation. To perform the simulation, we placed four bases in the same domain with each HCH isomer by ChemBioOffice2010 (Cambridge Soft, America). We analyzed the molecular orbital and attempted to identify the base that is most likely to bind to HCH. We also performed molecular dynamics using AMBER17^42^ with DNA.OL15^43^ force field and a customized force field for the HCHs with Antechamber^44^. The simulation details are described in Supplementary Methods.

### Reporting summary

Further information on experimental design is available in the Nature Research Reporting Summary linked to this article.

## Supplementary information

Supplementary Information

Reporting Summary

## Data Availability

The atomic coordinates and structure factors are deposited in the Protein Data Bank (www.pdb.org) with ID codes 2B0K (B-DNA). All other data supporting this study are available within the article and its Supplementary Information file, or from the authors upon reasonable request.
