# Supplementary material for: Organochlorinated pesticides expedite the enzymatic degradation of DNA
Source: Commun Biol. 2019 Feb 26;2:81. doi: 10.1038/s42003-019-0326-5 (PMC6391446; doi:10.1038/s42003-019-0326-5)
Supplement: Supplementary file 1 — Supplementary Information [file 42003_2019_326_MOESM1_ESM.docx]

**Supplementary Figures**


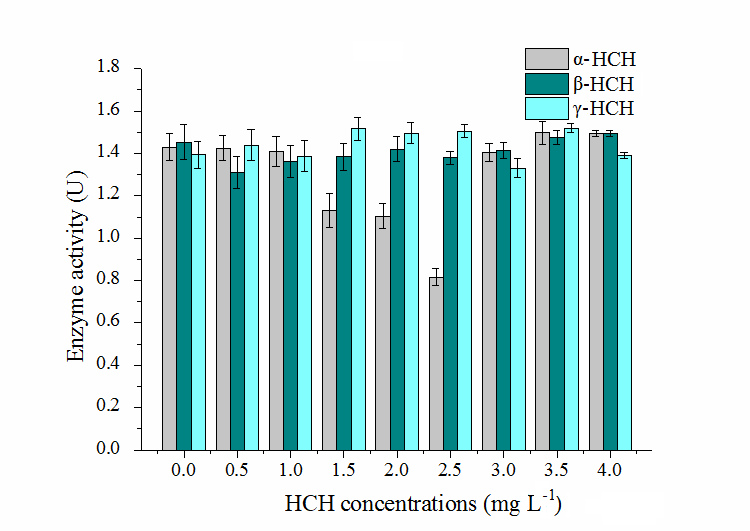


**Supplementary Figure 1.** Enzymatic activity of DNase I in the presence of 0, 0.5, 1, 1.5, 2, 2.5, 3, 3.5, and 4 mg L^-1^ HCH


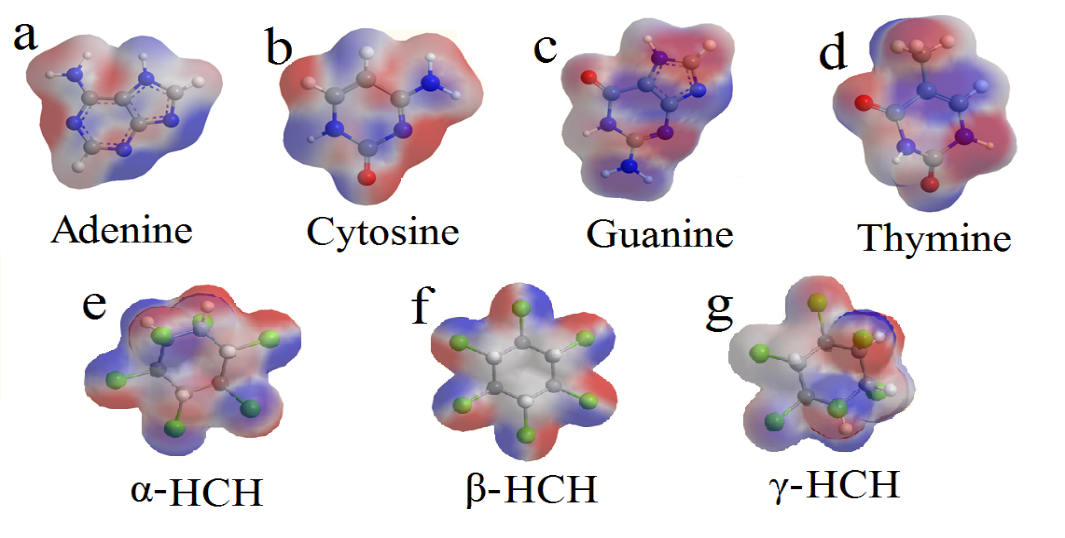


**Supplementary Figure 2.** Electrostatic potential distribution of four DNA bases and three HCH isomers: (**a**) adenine, (**b**) cytosine, (**c**) guanine, (**d**) thymine, (**e**) α-HCH, (**f**) β-HCH and (**g**) γ-HCH


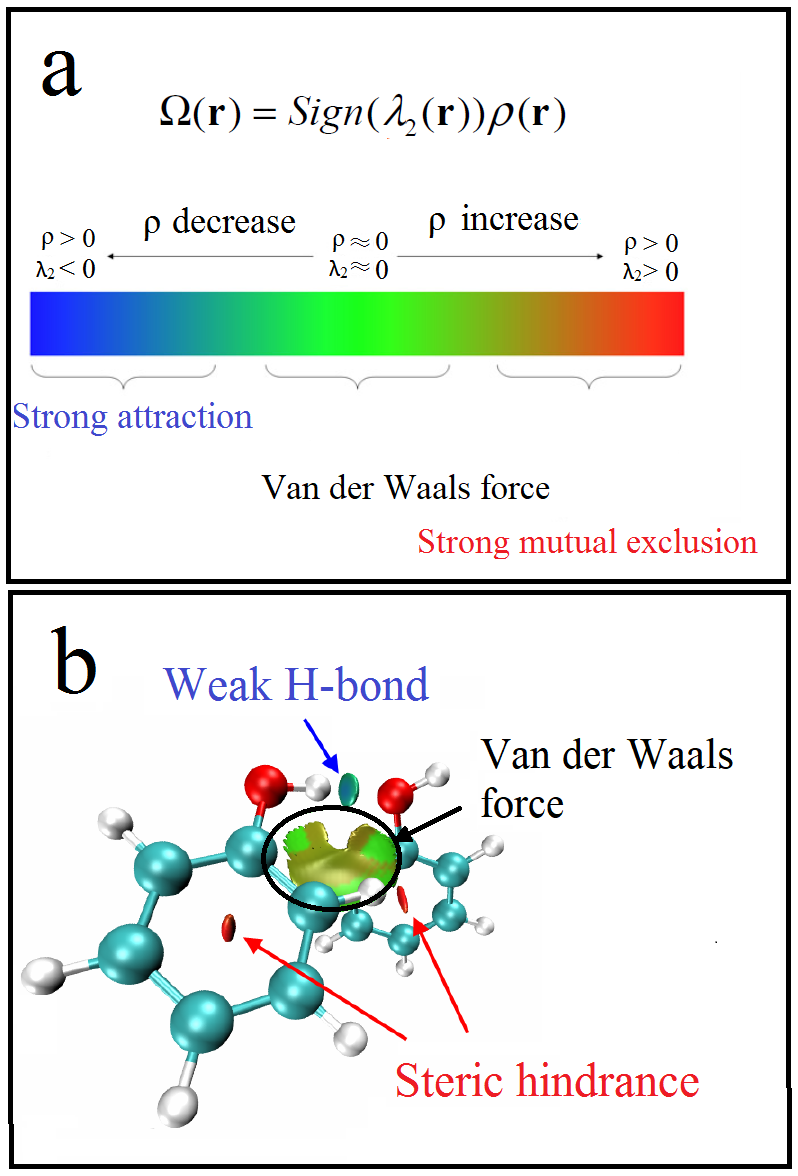


**Supplementary Figure 3.** Color scheme of (**a**) weak interaction isosurface according to the values of sign(λ_2_)ρ, and (**b**) gradient isosurface of benzene-benzene. ρ(**r**) represents the electron density function; Sign(λ_2_(**r**)) represents the second eigenvalue of the electron density Hessian matrix; and Ω(**r**) represents the reduced density gradient function


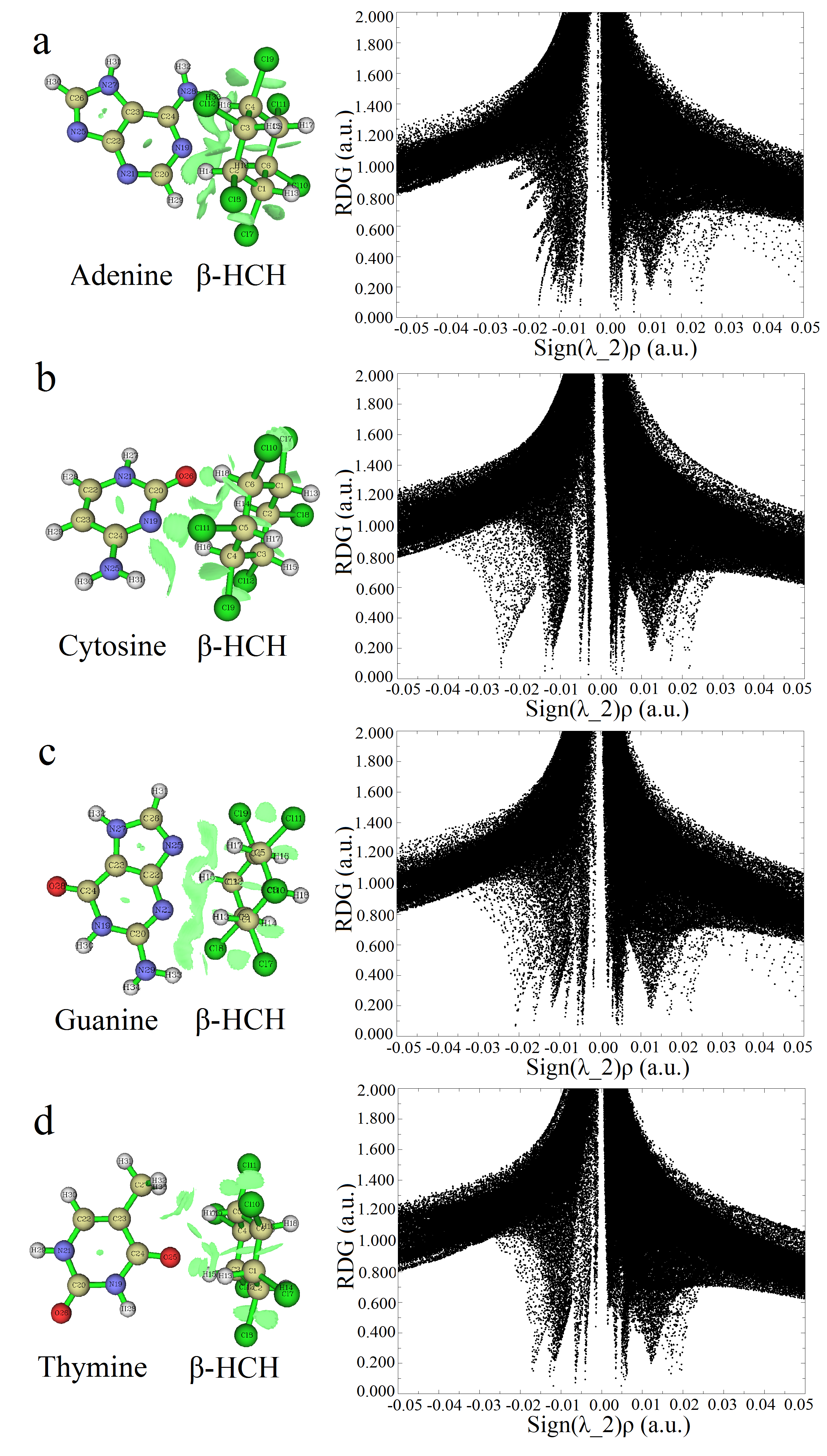


**Supplementary Figure 4.** Computational results of β-HCH binding to bases. Representative image of the gradient isosurface (left), the corresponding plots of reduced density gradient versus the sign of the second Hessian eigenvalues (right) of (**a**) adenine–β-HCH, (**b**) cytosine–β-HCH, (**c**) guanine–β-HCH, (**d**) thymine–β-HCH. The surfaces are colored on a blue-green-red scale according to the sign(λ_2_)ρ values (range −0.05 to 0.05 a.u.). Green areas between molecules indicate a weak Van der Waals force


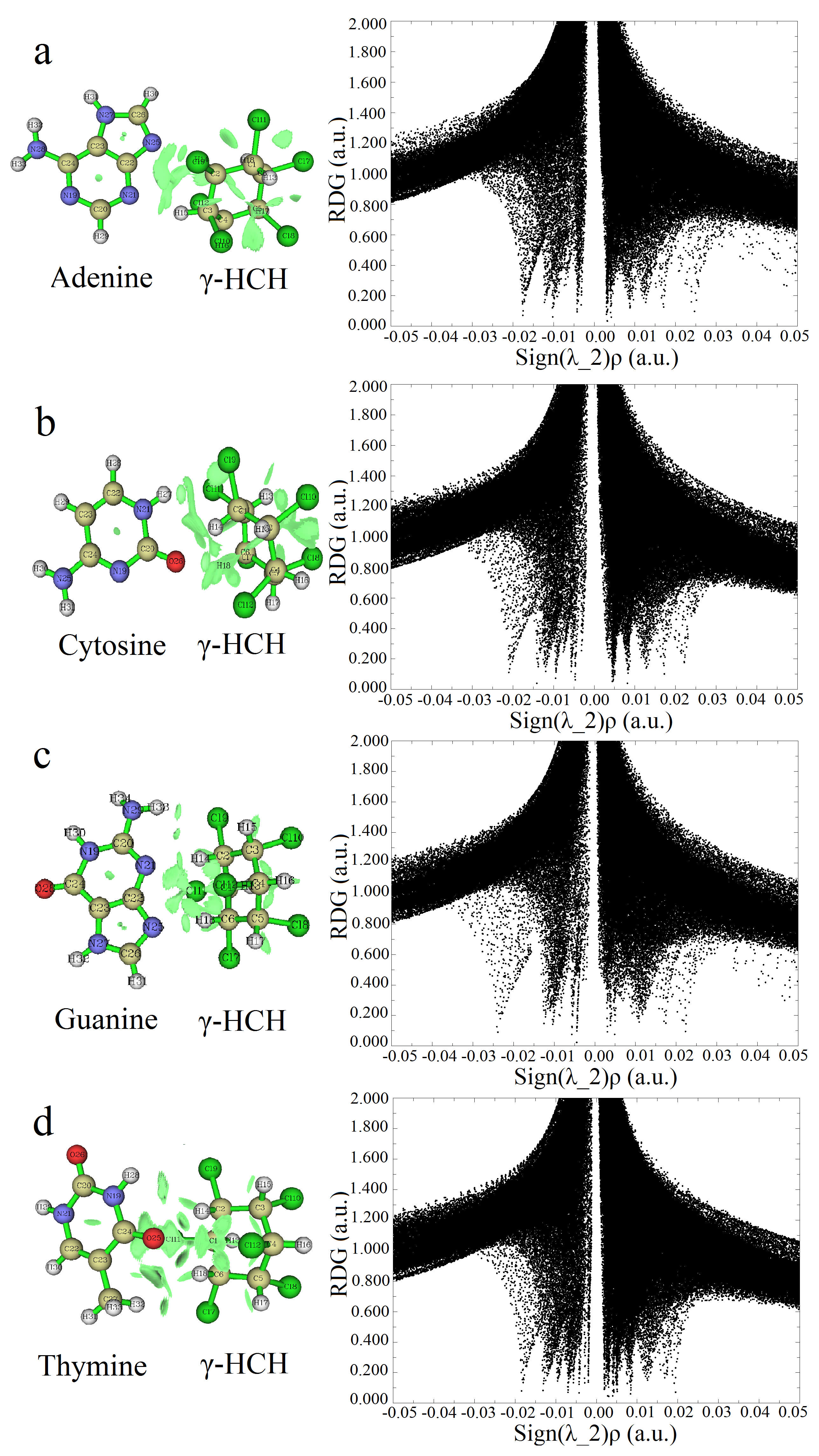


**Supplementary Figure 5.** Computational results of γ-HCH binding to bases. Representative image of the gradient isosurface (left), the corresponding plots of reduced density gradient versus the sign of the second Hessian eigenvalues (right) of (**a**) adenine–γ-HCH, (**b**) cytosine–γ-HCH, (**c**) guanine–γ-HCH, and (**d**) thymine–γ-HCH. The surfaces are colored on a blue-green-red scale according to the sign(λ_2_)ρ values (range −0.05 to 0.05 a.u.). Green areas between molecules indicate a weak Van der Waals force

**
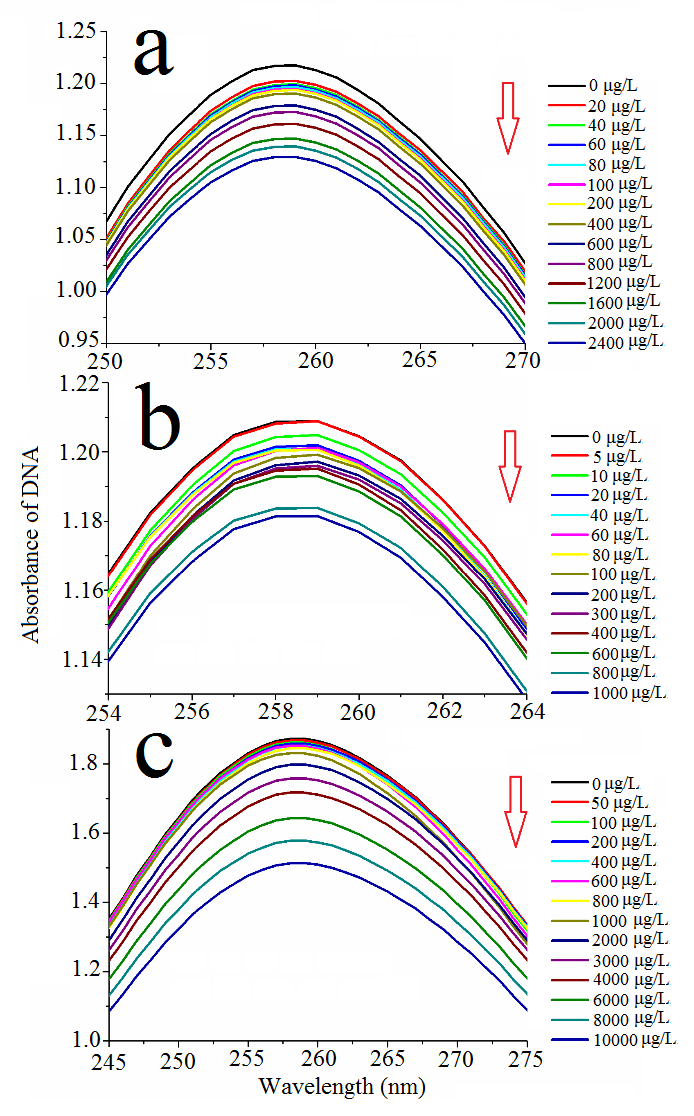
**

**Supplementary Figure 6.** UV-Vis spectra of DNA in solution in the presence of (**a**) α-HCH, (**b**) β-HCH and (**c**) γ-HCH at concentrations of 0−2400 μg L^-1^, 0−1000 μg L^-1^, 0−10000 μg L^-1^, respectively. The red arrow represents the decrease in absorbance with increasing HCH concentrations

|  | Binding Energy (kcal mol^-1^) | Binding Energy (eV) |
| --- | --- | --- |
| α-HCH–adenine | -15.7279 | -0.68382 |
| α-HCH–cytosine | -9.5766 | -0.41637 |
| α-HCH–guanine | -18.2779 | -0.79469 |
| α-HCH–thymine | -8.3687 | -0.36386 |
| β-HCH–adenine | -14.0488 | -0.61082 |
| β-HCH–cytosine | -6.3311 | -0.27527 |
| β-HCH–guanine | -18.0047 | -0.78281 |
| β-HCH–thymine | -8.2654 | -0.35937 |
| γ-HCH–adenine | -13.3531 | -0.58057 |
| γ-HCH–cytosine | -7.4683 | -0.32471 |
| γ-HCH–guanine | -14.5417 | -0.63225 |
| γ-HCH–thymine | -7.2568 | -0.31551 |

**Supplementary Table 1.** Calculated binding energies between HCHs and the bases of DNA.

| Absorption bands (cm^-1^) | Functional groups | References |
| --- | --- | --- |
| 970 ~ | DNA backbone or asymmetric PO^2-^ | [1](#_ENREF_1) |
| 1053 | Stretch vibration of P-O or C-O | [2](#_ENREF_2) |
| 1080 | Symmetrical stretch vibration of phosphate functional groups | [3](#_ENREF_3), [4](#_ENREF_4) |
| 1236 | Cytosine | [3](#_ENREF_3) |
| 1369 | Guanine | [3](#_ENREF_3), [4](#_ENREF_4) |
| 1420 and 1485 | DNA structure | [2](#_ENREF_2), [5](#_ENREF_5), [6](#_ENREF_6), [7](#_ENREF_7) |
| 1531 | Imidazole ring | [8](#_ENREF_8), [9](#_ENREF_9) |
| 1649 | Stretch of C=C or C=N in base | [8](#_ENREF_8), [9](#_ENREF_9) |
| 1691 | Guanine carbonyl vibration | [10](#_ENREF_10), [11](#_ENREF_11), [8](#_ENREF_8), [12](#_ENREF_12) |

**Supplementary Table 2.** The absorption bands in the FTIR spectra of DNA and their corresponding functional groups.

| Absorption bands (cm^-1^) | Functional groups | References |
| --- | --- | --- |
| 400-1000 | unsaturated bands | [13](#_ENREF_13) |
| 1404, 1297 | amide III (N–H bending and C–N stretching vibrations) | [13](#_ENREF_13), [14](#_ENREF_14) |
| 1544, 1462 | amide II (C-N+N-H) | [12](#_ENREF_12), [13](#_ENREF_13) |
| 1632 | amide I (C(N)=O) of α-helix of DNase I | [12](#_ENREF_12), [13](#_ENREF_13) |

**Supplementary Table 3.** The absorption bands in the FTIR spectra of DNase I and their corresponding functional groups.

| HCH | Molecular Structures | Molecular Weight  (g mol^-1^) | Purity | Density  g (cm^3^) ^-1^ | Solubility (25 °C, μg L^-1^) |
| --- | --- | --- | --- | --- | --- |
| \| α-HCH \| \| --- \| | 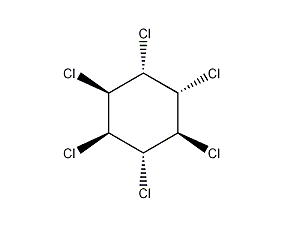 | 290.83 | 99% | 1.59 | 1630 |
| β-HCH | 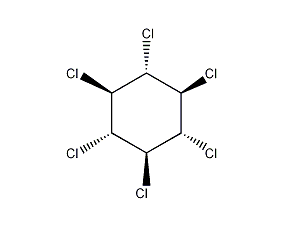 | 290.83 | 99% | 1.89 | 700 |
| γ-HCH |  | 290.83 | 99% | 1.87 | 7900 |

**Supplementary Table 4.** Molecular structures and physicochemical properties of α-HCH, β-HCH and γ-HCH.

**Supplementary Methods**

**Fluorescence quenching titration experiments.** Fluorescence quenching titration experiments were used to determine the binding of HCHs with DNA. First, the DNA working solution (10 mg L^-1^) was prepared by diluting 1 g L^-1^ DNA stock solution with 10 mmol L^-1^ Tris-HCl. Then, 100 μL of 1 g L^-1^ ethidium-bromide was added into 50 mL of 10 mg L^-1^ DNA working solution for the fluorescence labeling of the DNA. Afterward, 10 mg L^-1^ of HCH solution was gradually titrated into the ethidium-bromide-labeled DNA solution by a chromatographic injector with an injection volume of 5 μL. The obtained HCH concentrations were 0−15 μg L^-1^. After each addition of HCHs, the mixture was stirred for 20 min at 25 °C and 160 rpm on a magnetic stirrer to obtain a stable fluorescence intensity. Then 2 mL of the mixture was placed in a 4-mL quartz cuvette with a 1-cm path length. The fluorescence intensity was measured at excitation wavelength of 450−600 nm and emission wavelength of 550−650 nm with a 2 nm resolution by a fluorescence spectrophotometer (F-7000, Hitachi, Japan). The maximal fluorescence intensity was obtained at excitation/emission of 522 nm/604 nm for DNA-ethidium-bromide, and the peak fluorescence intensity was averaged from three measurements. The titration experiments were conducted in duplicates. T-test was used for statistical analyses. Peak fluorescence intensity versus the quencher (HCH) concentrations was described with the Stern-Volmer equation (1):

$\frac{\text{F}_{\text{0}}}{\text{F}}\text{=1+}\text{K}_{\text{q}}\text{τ}_{\text{0}}\left[ \text{Q} \right]\text{=1+}\text{K}_{\text{SV}}\text{[}\text{Q}\text{]}$ (1)


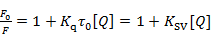

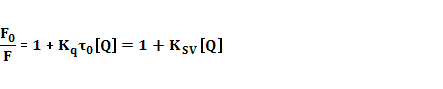


where *F*_0_ and *F* are the DNA-ethidium-bromide fluorescence intensity before and after quenching, *K*_q_ is the bimolecular quenching rate constant, *τ*_0_ is the average lifetime of the fluorophore in the absence of quencher, [*Q*] is the concentration of the quencher, and *K*_sv_ is the quenching constant. For a static quenching process, the binding constant (*K*_A_) and the number of binding sites (n) were calculated by the following equation (2):

$\text{Log}\left[ \frac{\text{F}_{\text{0}}\text{-}\text{F}}{\text{F}} \right]\text{=Log}{\text{ }\text{K}}_{\text{A}}\text{+}\text{ }\text{n}\text{ Log}\text{ }\text{[}\text{Q}\text{]}$ (2)


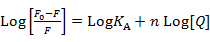


**FTIR Analysis.** Fourier transform infrared spectroscopy (FTIR) was used to infer the binding mechanisms of HCHs with DNA. To obtain sufficient amount of DNA samples for these analyses, 10 mL of DNA suspension was reproduced following the experimental condition identical to that of the DNA degradation experiments (i.e., 100 mg L^-1^ DNA, 4 mg L^-1^ HCH, 0.7 U μL^-1^ DNase I, initial pH 7.0, 37 °C), and then freeze-dried at −65 °C for one week in a freeze-dryer (Beta 1-8 LD plus, Christ, Osterode, Germany). The DNA samples without DNase I were similarly prepared. The freeze-dried DNA samples were mixed with potassium bromide (KBr) at a ratio of 1:100. Then, the mixtures were ground into fine powder using a carnelian mortar. Finally, the disc-shaped samples were analyzed by a Nicolet NEXUS870 FTIR spectrometer (Thermo Scientific, USA).

**Molecular dynamics simulations.** Docking analysis was first performed using AutoDock^[15](#_ENREF_15)^ to explore the stable state of HCH and DNA using a Dell Computer (Ubuntu Linux 17.04, Intel(R) Celeron(R) CPU G5140, 2.80 GHz, 4 GB memory). The initial structure of B-DNA dodecamer d(CGCGAATTCGCG)_2_ was downloaded from the Protein Data Bank (PDBID: 2B0K). The geometry of HCH was drawn by ChemBioDraw Ultra 14.0. The optimized geometries of DNA and HCHs were calculated by Gaussian 09 using the ωB97XD/6-311G** basis sets. Non-polar hydrogen atoms were merged, and Kollman charges were added using MGL Tools-1.5.6[^16^](#_ENREF_16). The post-docking analysis was performed using Chimera 1.11.2 and Discovery Studio 2016 Client. The molecular modeling is based on the result of docking. Then, we performed the simulation of molecular dynamics using[^17^](#_ENREF_17) with DNA.OL15[^18^](#_ENREF_18) force field and a customized force field for the HCHs with Antechamber[^19^](#_ENREF_19). The systems were placed in a cubic box with periodic boundary conditions and solvated with specific TIP3P model water molecules. Due to the negative charge of DNA, Na^+^ was added to the system as counterion. The structural properties were calculated using the Cpptraj module of AMBER17. The root mean square deviation (RMSD) was calculated by the following equation:

$$\text{RMSD=}\sqrt{\frac{\text{1}}{\text{N}_{\text{atm}}}\sum_{\text{A}}^{\text{N}_{\text{atm}}} {\text{(}\text{r}_{\text{A}}\text{-}\text{r}_{\text{A}}^{\text{ref}}\text{)}}^{\text{2}}}$$

where *N*_atm_ is the number of atoms in the selected range, **r** is the position of atom, *A* is the atomic number, and ref is the superscript representing the reference structure. Dynamic simulation results are analyzed using Visual Molecular Dynamics 1.9.3 and Origin 8.5.1.

**Supplementary References**

1. Mao, Y., Daniel, L. N., Whittaker, N. & Saffiotti, U. DNA binding to crystalline silica characterized by Fourier-transform infrared spectroscopy. *Environ. Health Persp.* **102**, 165-171 (1994).

2. Alex, S. & Dupuis, P. FTIR and Raman investigation of cadmium binding by DNA. *Inorg. Chim. Acta* **157**, 271-281 (1989).

3. Cai, P., Huang, Q. Y. & Zhang, X. W. Interactions of DNA with clay minerals and soil colloidal particles and protection against degradation by DNase. *Environ. Sci. Technol.*  **40**, 2971-2976 (2006).

4. Tsuboi, M. Application of Infrared Spectroscopy to Structure Studies of Nucleic Acids. *Appl. Spectrosc. Rev.* **3**, 45-90 (1970).

5. Parker, A. W. & Quinn, S. J. Infrared Spectroscopy of DNA. *Encyclopedia of Biophysics* (2013).

6. Tajmir-Riahi, H. A., Neault, J. F. & Naoui, M. Does DNA acid fixation produce left-handed Z structure? *Febs Letters* **370**, 105-108 (1995).

7. Lindqvist, M. & Gräslund, A. An FTIR and CD study of the structural effects of G-tract length and sequence context on DNA conformation in solution. *J. Mol. Biol.* **314**, 423-432 (2001).

8. Banyay, M. & Gräslund, A. Structural Effects of Cytosine Methylation on DNA Sugar Pucker Studied by FTIR. *J. Mol. Biol.* **324**, 667-676 (2002).

9. Taillandier, E., Peticolas, W. L., Adam, S., Huynh-Dinh, T. & Igolen, J. Polymorphism of the d(CCCGCGGG)_2_ double helix studied by FTIR spectroscopy. *Spectrochim. Acta A* **46**, 107-112 (1990).

10. Ouameur, A. A. & Tajmir-Riahi, H. A. Structural analysis of DNA interactions with biogenic polyamines and cobalt(III)hexamine studied by Fourier transform infrared and capillary electrophoresis. *J. Biol. Chem.* **279**, 42041-42054 (2004).

11. Loprete, D. M. & Hartman, K. A. Conditions for the stability of the B, C, and Z structural forms of poly(dG-dC) in the presence of lithium, potassium, magnesium, calcium, and zinc cations. *Biochemistry* **32**, 4077-4082 (1993).

12. Tajmirriahi, H. A., N'Soukpoékossi, C. N. & Joly, D. Structural analysis of protein–DNA and protein–RNA interactions by FTIR, UV-visible and CD spectroscopic methods. *Spectro. An Inter. J.* **23**, 81-101 (2009).

13. Haris, P. I. & Severcan, F. FTIR spectroscopic characterization of protein structure in aqueous and non-aqueous media. *J. Mol. Catal. B-Enzym.* **7**, 207-221 (1999).

14. Andreoni, V. et al. Bacterial communities and enzyme activities of PAHs polluted soils. *Chemosphere* **57**, 401-412 (2004).

15. Morris, G. M. et al. AutoDock4 and AutoDockTools4: Automated docking with selective receptor flexibility. *J. Comput. Chem.* **30**, 2785-2791 (2009).

16. Morris, G. M. et al. Automated docking using a Lamarckian genetic algorithm and an empirical binding free energy function. *J. Comput. Chem.* **19**, 1639-1662 (1998).

17. A. Case, D. S. C., T. E. Cheatham, III, T.A. Darden, R. E. Duke, T. J. Giese, H. Gohlke, A. W. Goetz, D. Greene, N. Homeyer, S. Izadi, A. Kovalenko, T. S. Lee, S. LeGrand, P. Li, C. Lin, J. Liu, T. Luchko, R. Luo, D. Mermelstein, K. M. Merz, G. Monard, H. Nguyen, I. Omelyan, A. Onufriev, F. Pan, R. Qi, D. R. Roe, A. Roitberg, C. Sagui, C. L. Simmerling, W. M. Botello-Smith, J. Swails, R. C. Walker, J. Wang, R. M. Wolf, X. Wu, L. Xiao, D. M. York and P. A. Kollman AMBER 2017. *University of California, San Francisco* (2017).

18. Galindomurillo, R. et al. Assessing the Current State of Amber Force Field Modificationsfor DNA. *J. Chem. Theory. Comput.* **12**, 4114-4127 (2016).

19. Wang, J., Wang, W., Kollman, P.A. & Case, D.A. Automatic atom type and bond type perception in molecular mechanical calculations. *J. Mol. Graph. Model.* **25**, 247-260 (2006).
